# Biosynthesis of Piceatannol from Resveratrol in Grapevine Can Be Mediated by Cresolase-Dependent *Ortho*-Hydroxylation Activity of Polyphenol Oxidase

**DOI:** 10.3390/plants13182602

**Published:** 2024-09-18

**Authors:** Ascensión Martínez-Márquez, Susana Selles-Marchart, Hugo Nájera, Jaime Morante-Carriel, Maria J. Martínez-Esteso, Roque Bru-Martínez

**Affiliations:** 1 Plant Proteomics and Functional Genomics Group, Department of Biochemistry and Molecular Biology and Soil Science and Agricultural Chemistry, Faculty of Science, University of Alicante, 03690 Alicante, Spain; susana.selles@ua.es (S.S.-M.); hnajera@cua.uam.mx (H.N.); jaime.morante@ua.es (J.M.-C.); mjose.martinez@ua.es (M.J.M.-E.); roque.bru@ua.es (R.B.-M.); 2 Alicante Institute for Health and Biomedical Research (ISABIAL), 03010 Alicante, Spain; 3 Research Technical Facility, Proteomics and Genomics Division, University of Alicante, San Vicente del Raspeig, 03690 Alicante, Spain; 4 Departamento de Ciencias Naturales, Universidad Autónoma Metropolitana–Cuajimalpa, Av. Vasco de Quiroga 4871, Colonia Santa Fe Cuajimalpa, Alcaldía Cuajimalpa de Morelos, Mexico City 05348, Mexico; 5 Plant Biotechnology Group, Faculty of Forestry and Agricultural Sciences, Quevedo State Technical University, Av. Quito km. 1 1/2 vía a Santo Domingo de los Tsachilas, Quevedo 120501, Ecuador; 6 Multidisciplinary Institute for the Study of the Environment (IMEM), University of Alicante, 03690 Alicante, Spain

**Keywords:** piceatannol, polyphenol oxidase (PPO), cresolase activity, resveratrol, *Vitis vinifera*

## Abstract

Piceatannol is a naturally occurring hydroxylated analogue of the stilbene phytoalexin resveratrol that can be found in grape fruit and derived products. Piceatannol has aroused great interest as it has been shown to surpass some human health-beneficial properties of resveratrol including antioxidant activity, several pharmacological activities and also bioavailability. The plant biosynthetic pathway of piceatannol is still poorly understood, which is a bottleneck for the development of both plant defence and bioproduction strategies. Cell cultures of *Vitis vinifera* cv. Gamay, when elicited with dimethyl-β-cyclodextrin (MBCD) and methyl jasmonate (MeJA), lead to large increases in the accumulation of resveratrol, and after 120 h of elicitation, piceatannol is also detected due to the regiospecific hydroxylation of resveratrol. Therefore, an *ortho*-hydroxylase must participate in the biosynthesis of piceatannol. Herein, three possible types of resveratrol hydroxylation enzymatic reactions have been tested, specifically, a reaction catalyzed by an NADPH-dependent cytochrome, P450 hydroxylase, a 2-oxoglutarate-dependent dioxygenase and *ortho*-hydroxylation, similar to polyphenol oxidase (PPO) cresolase activity. Compared with P450 hydoxylase and the dioxygenase activities, PPO displayed the highest specific activity detected either in the crude extract, the particulate or the soluble fraction obtained from cell cultures elicited with MBCD and MeJA for 120 h. The overall yield of PPO activity present in the crude extract (107.42 EU) was distributed mostly in the soluble fraction (66.15 EU) rather than in the particulate fraction (3.71 EU). Thus, partial purification of the soluble fraction by precipitation with ammonium sulphate, dialysis and ion exchange chromatography was carried out. The soluble fraction precipitated with 80% ammonium sulphate and the chromatographic fractions also showed high levels of PPO activity, and the presence of the PPO protein was confirmed by Western blot and LC-MS/MS. In addition, a kinetic characterization of the cresolase activity of partially purified PPO was carried out for the resveratrol substrate, including Vmax and Km parameters. The Km value was 118.35 ± 49.84 µM, and the Vmax value was 2.18 ± 0.46 µmol min^−1^ mg^−1^.

## 1. Introduction

**Figure 1:**
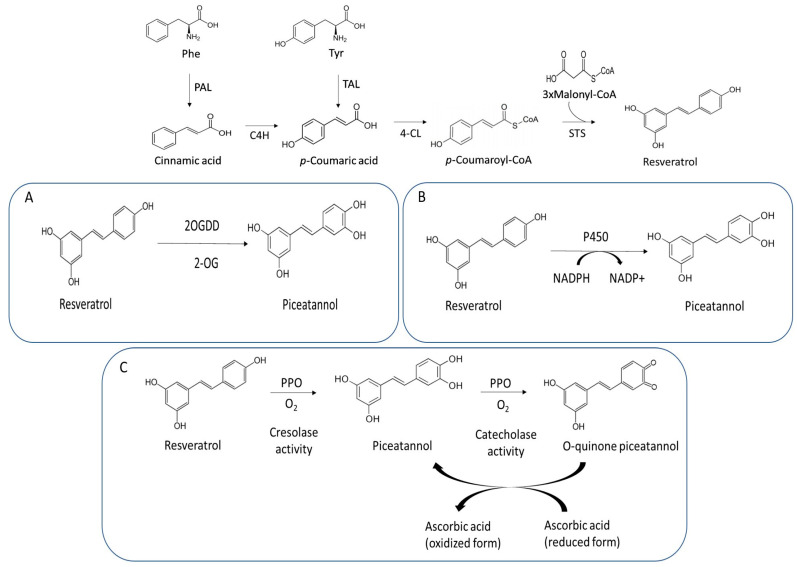
Biosynthesis pathway of resveratrol and piceatannol. The conversion of *trans*-resveratrol to piceatannol can be catalyzed by 2-oxoglutarate-dependent dioxygenase enzyme in the presence of 2-oxoglutarate (**A**), P450 enzyme in the presence of NADPH (**B**) and PPO in the presence of ascorbic acid (**C**). TAL: tyrosine ammonia lyase, PAL: phenylalanine ammonia lyase, 4-CL: 4-coumaroyl-CoA ligase, C4H: coumarate 4-hydroxylase, STS: stilbene synthase, 2-OGDD: 2-oxoglutarate-dependent dioxygenase, P450: cytochrome P450, PPO: polyphenol oxidase.

Stilbenes are a restricted group of the major class of plant secondary metabolites called phenolic compounds, having a characteristic 1,2-diphenylethylene core. Unlike other phenolics, stilbenes occur in a few phylogenetically unrelated seed plant families [1] due to the independent evolution of chalcone synthases which gives rise to stilbene synthases (STSs) [2]. STSs catalyze a diverting reaction following the phenylpropanoid pathway from L-phenylalanine to a CoA-activated phenylpropanoid that accepts three malonyl CoA units to produce the stilbene core and releasing three CO_2_ molecules (Figure 1). In grapevine (*Vitis vinifera* L.), the phenylpropanoid acceptor is p-coumaroyl CoA, thus producing trans-resveratrol (3,4′,5-trihydroxy-*trans*-stilbene) (*t*-R) [3,4], and thus making STS to compete with flavonoid biosynthesis for the same precursor.

For most polyphenol classes, their structural diversity arises from the action of a set of enzymes, including glycosyltransferases, methyltransferases, peroxidases, prenyltransferases, hydroxylases or laccases, on a structural core [5]. For the stilbene family, the starting structure is resveratrol, whose biosynthetic pathway is well known, but most enzymatic steps in the obtainment of resveratrol derivatives such as piceatannol remain to be characterized and even identified. Up to 78 resveratrol derivatives have been described in grapevine vegetative tissues [6], including piceatannol [7]; its monomethylated form, isorhapontigenin; and glycosylated forms such as astringin, rhaponticin and isorhapontin [8,9].

Like resveratrol, piceatannol has antioxidative and anti-inflammatory activities that have an array of health-beneficial effects, including anticarcinogenic effects in different stages of tumour generation and progression, as well as other activities preventing metabolic and inflammatory diseases such as anti-obesity, antidiabetic, cardioprotective, neuroprotective, anti-allergic and anti-ageing properties [10,11]. Thus, piceatannol has aroused great interest as it has been shown to even surpass some resveratrol properties, such as antioxidant activity [12], pharmacological activity [13] and bioavailability [14].

Hydroxylated resveratrol derivatives, such as piceatannol or oxyresveratrol, are supposed to result from the action of hydroxylases. Resveratrol hydroxylation to produce piceatannol has been reported for the cytochrome P450-dependent hydroxylases human CYP1B1 [15,16], CYP1A1/2 [17] and the bacterial CYP102A1 [18]. However, it has been recently shown that in *Morus alba*, a 2-hydroxylation reaction occurs on the acceptor p-coumaroyl CoA upstream the STS reaction; thus, the product oxyresveratrol is synthesized in a parallel pathway to resveratrol and does not derive from it [19].

The plant biosynthetic pathway of piceatannol is still poorly understood. In *Picea* spp., three possible pathways have been proposed for the formation of piceatannol involving either a double, 3′, 4′, hydroxylation of the core stilbene pinosylvin, the 3′-hydroxylation of resveratrol or the alternative use of the o-diphenol caffeoyl-CoA as the phenylpropanoid acceptor by STS [20]. In vitro enzyme assays with the STS from *Picea* spp. showed that this enzyme uses only *p*-coumaroyl-CoA as a substrate, thus excluding the hypothesis of a direct formation of piceatannol from caffeoyl-CoA in this plant [21]. In *Vitis* spp., pinosylvin has not been identified [22], in contrast to piceatannol [7] and its methylated and glycosylated derivatives [8,9]. These facts give way to the hypotheses of the involvement of 3′-hydroxylase or *ortho*-hydroxylase in the biosynthesis of piceatannol in grapevine; however, to our knowledge, there are no reports on grapevine-specific hydroxylases involved in the direct hydroxylation of resveratrol [4]. The occurrence of piceatannol in grapevine tissues and its accumulation in cell cultures treated with elicitors dimethyl-β-cyclodextrin (MBCD) and methyl jasmonate (MeJA) [16] led us to seek candidate enzymes that might catalyze the formation of piceatannol. Besides the scientific interest in characterizing stilbene metabolism in grapevine, finding the enzyme responsible for piceatannol biosynthesis and its encoding gene(s) would open up new ways to sustainably produce this antitumoral compound through the design of metabolic engineering and synthetic biology approaches in similar ways as for other resveratrol derivatives [16]. Thus, using a reverse genetics strategy, we considered three types of resveratrol hydroxylation reactions, namely, an NADPH-dependent cytochrome P450 hydroxylase similar to human CYP1B1 [15]; a 2-oxoglutarate-dependent dioxygenase similar to flavonoid hydroxylating enzymes -flavanone 3′ hydroxylase, flavonol synthase or anthocyanidin synthase [23]; and a cofactor-independent *ortho*-hydroxylation similar to polyphenol oxidase (PPO) cresolase activity [24] (Figure 1A–C). Here, we showed the last reaction type, cresolase activity of PPO, as the most likely pathway of piceatannol biosynthesis, performed a partial purification, detected the protein by Western blotting in the high-activity fractions and performed a kinetic characterization of resveratrol conversion into piceatannol. Using an LC-MS proteomics workflow, full-length PPO isoforms were identified in the active samples and proposed as most likely responsible for this function in grapevine.

## 2. Materials and Methods

### 2.1. Plant Material

*Vitis vinifera* L. cv. Gamay calli were kindly supplied by Drs. J. C. Pech and A. Latché (ENSA, Toulouse, France) in 1989. This cell line was maintained as both solid and liquid cultures in Gamborg B5 medium, as described elsewhere [25].

### 2.2. Elicitor Treatments

Elicitor treatments were carried out as previously described [25,26,27]. Briefly, a weighted amount of filtered and washed cells was transferred into shaking flasks and suspended in fresh growth medium (4 mL/g of cell FW) supplemented with both 50 mM MBCD and 0.1 mM MeJA as elicitors. The cell suspension was incubated with continuous rotary shaking (100 rpm) at 25 °C and under a 16 h light/8 h dark photoperiod for 120 h.

### 2.3. Determination of Stilbenoids

Samples of extracellular and intracellular stilbenes of *Vitis* cell culture were prepared as described [16]. Briefly, 3 volumes of extracellular medium were vortexed for 1 min with 1 volume of ethyl acetate followed by accelerated phase separation in a benchtop microcentrifuge and recovery of the organic phase. The extraction was repeated and organic phases were pooled. The solvent was removed in a speed vac centrifuge and the solid residue redissolved in 1 volume of 80% *v*/*v* methanol. Targeted quantitative analysis of stilbenoids was performed by MRM using external calibration curves with authentic compounds by monitoring quantifier transitions 229/107 for resveratrol and 245/135 for piceatannol in an Agilent 1290 Infinity UHPLC coupled to an Agilent 6490 QQQ mass spectrometer (Agilent Technologies Inc., Santa Clara, CA, USA) as described in [28].

### 2.4. Preparation of Grapevine Cells Subcellular Fractions and Protein Extracts

Grapevine cells elicited with 50 mM MBCD and 0.1 mM MeJA were harvested after 120 h incubation from liquid cell cultures by filtration under a gentle vacuum. A cell homogenate was obtained after mechanical lysis of cell suspensions in a Potter-Elvehjem homogenizer in extraction buffer (50 mM HEPES pH 7.5, 0.25 M sucrose, 1% (*w*/*v*) PVPP, 5% (*w*/*v*) glycerol, 10 mM EDTA, 10 mM Na_2_O_5_S_2_, 10 mM ascorbic acid, 1 mM PMSF and 0.065% *v*/*v* reconstituted Sigma Protease inhibitor cocktail (ref. P8465) (Merck KGaA, Darmstadt, Germany)) at a ratio of 2 mL per gram of plant material at 4 °C. Cellular debris was removed by centrifugation at 8000× *g* for 10 min at 4 °C and the supernatant (crude extract, Ec) ultracentrifuged at 100,000× *g* for 1:30 h at 4 °C. The supernatant (soluble fraction, Fs) was kept apart and the pellet (particulate fraction, Fp) was washed twice by resuspension in double volume of washing buffer (50 mM HEPES pH 7.5, 5% (*w*/*v*) glycerol, 10 mM ascorbic acid, 1 mM PMSF and 0.065% *v*/*v* Sigma Protease inhibitor cocktail), and after recovery by ultracentrifugation as above, the washed pellet was resuspended in 15 mL of washing buffer.

The soluble fraction was precipitated sequentially with ammonium sulphate at 20%, 45% and 80% saturation. The pellet obtained from the precipitation with 80% ammonium sulphate was resuspended in 10 mL of washing buffer, dialyzed to desalt and to eliminate molecules smaller than 10 kDa and applied onto a Hi PrepTm 16/10 DEAE FF (Amersham Biosciences, Uppsala, Sweden) column, equilibrated with 50 mM HEPES pH 7.5, 5% glycerol. The column was washed with the same buffer and then eluted with a step gradient of NaCl (0.0–1.0 M) in the same buffer. The fractions were stored at −80 °C for enzyme assays.

### 2.5. Enzymatic Hydroxylation Assay

Enzyme preparations used for the assay were either crude extract, soluble native protein after precipitation with 80% ammonium sulphate, particulate fractions or different chromatographic fractions. The enzyme assays were performed in a final volume of 0.5 mL containing 200 µL of enzyme preparation, 200 µM of *t*-R, 0.1 M potassium phosphate buffer pH 7.5 and 1 mM ascorbate as fixed components, and 1 mM NADPH, 1 mM oxoglutarate and 10 mM sodium metabisulfite as variable components, depending on the type of hydroxylating enzymatic reaction sought. The reactions were incubated for 3 h at room temperature. After 3 h, reaction products were extracted with 25% (*v*/*v*) ethyl acetate and analyzed by MRM as described above.

To determine the kinetic parameters of PPO, 5 to 200 µM of *t*-R was used. The data were fit to the standard Michaelis–Menten equation.

### 2.6. Protein Identification by Liquid Chromatography–Tandem Mass Spectrometry and Database Search

Aliquots of the crude extract, the soluble fraction precipitated with 80% ammonium sulphate, the particulate fraction and different chromatographic fractions were treated for protein precipitation as described in [29], with slight modifications. Briefly, the sample was brought to a volume of 750 µL by adding distilled water followed by 8.5 µL of 2% (*w*/*v*) sodium deoxycholate solution [30]; after mixing and incubation on ice for 10 min, 250 µL of 24% (*w*/*v*) trichloroacetic acid (TCA) was added, vortexed and incubated for 30 min on ice to quantitatively precipitate proteins. The protein pellet obtained by centrifugation at 14,000× *g* for 10 min at 4 °C was washed twice with chilled 10% (*w*/*v*) TCA in acetone followed by being washed twice in pure chilled acetone. Finally, the clean protein precipitate obtained was left to dry at room temperature, solubilized in 6 M urea, and quantified by an RC DC protein assay (BIO-RAD, Hercules, CA, USA) [31]. One hundred micrograms of precipitated protein sample was reduced, alkylated with iodoacetamide and digested with trypsin as previously described [32], and 30 µg of the resulting peptides was desalted with PepClean C-18 Spin Columns (Agilent Technologies, Santa Clara, CA, USA) according to the manufacturer’s recommendations.

LC-MS/MS analyses were performed using an Agilent 1290 Infinity UHPLC coupled through an Agilent Jet Stream^®^ interface to an Agilent 6550 iFunnel Q-TOF mass spectrometer (Agilent Technologies) system. Peptides were separated in a reverse-phase Agilent AdvanceBio Peptide mapping column (2.1 mm × 250 mm, 2.7 μm particle size, operated at 50 °C) using a 140 min linear gradient of 3–40% ACN in 0.1% formic acid at 0.400 mL/min flow rate. The mass spectrometer was operated in high-sensitivity mode. Source parameters employed gas temperature (250 °C), drying gas (14 L/min), nebulizer (35 psi), sheath gas temp (250 °C), sheath gas flow (11 L/min), capillary voltage (3500 V), and a fragmentor (360 V). The data were acquired in positive-ion mode with Agilent MassHunter Workstation Software, LC/MS Data Acquisition B.08.00 (Build 8.00.8058.0) operating in Auto MS/MS mode, whereby the 20 most intense ions (charge states, 2–5) within the 300–1700 *m*/*z* mass range above a threshold of 1000 counts were selected for MS/MS analysis. MS/MS spectra (50–1700 *m*/*z*) were collected with the quadrupole set to “narrow” resolution and were acquired until 25,000 total counts were collected or for a maximum accumulation time of 333 ms.

Each MS/MS spectrum was preprocessed with the extraction tool of Spectrum Mill Proteomics Workbench (Agilent) to obtain a peak list and to improve the spectral quality by merging MS/MS spectra with the same precursor (Δ *m*/*z* < 1.4 Da and chromatographic Δ t < 15 s). The reduced dataset was searched against the proteome database of UniProtKB *Vitis vinifera* and contaminant proteins in the identity mode with the MS/MS search tool of Spectrum Mill Proteomics Workbench and with the following settings: trypsin, up to 2 missed cleavages, the carbamidomet–hylation of Cys as fixed modifications, the oxidation of Met, the deamidation of Asn and Gln and pyroGlu as variable modification and mass tolerance of 20 ppm for precursor and 50 ppm for product ions. Peptide hits were filtered for a score ≥ 6 and percent score of peak intensity (%SPI) ≥ 60.

### 2.7. Western Blotting

Protein samples were resolved by SDS-PAGE and electro-transferred to the Hybond-P PVDF membranes (GE Healthcare, Chicago, IL, USA). Membranes were probed at 4 °C overnight with rabbit anti-loquat PPO antisera [33] at a 1:5000 dilution, followed by incubation at room temperature for 1 h with horseradish peroxidase-conjugated goat anti-rabbit IgG at a 1:10,000 dilution. Detection was performed by ECL using the Prime Western Blotting Detection Reagent SuperSignal West Dura system (GE Healthcare, Amersham, UK).

## 3. Results and Discussion

### 3.1. Determination of Resveratrol Hydroxylating Activity

It is well known that elicitation treatments of *V. vinifera* cv. Gamay cell cultures with MBCD combined with MeJA lead to large increases in the accumulation of t-R, and after 120 h of elicitation, piceatannol is also detected, likely due to the regiospecific hydroxylation of resveratrol [16]. The reported bioactivity [34] and biotechnological importance of piceatannol have led us to investigate its biosynthetic pathway, unknown until now, and we have taken the hypothesis that piceatannol is produced by the *o*-hydroxylation of resveratrol in grapevine. Samples were prepared sequentially, starting from a crude extract of grapevine cells obtained from elicited cultures fractionated by ultracentrifugation, ammonium sulphate precipitation and DEAE FF ion exchange chromatography, leading to the obtainment of different samples and fractions of decreasing complexity. Three types of resveratrol hydroxylation enzymatic reactions were tested, specifically, (1) a reaction catalyzed by a NADPH-dependent cytochrome, P450 hydroxylase, which contained NADPH and sodium metabisulfite as variable components of the reaction medium; (2) a reaction catalyzed by a 2-oxoglutarate-dependent dioxygenase which contained oxoglutarate and sodium metabisulfite as variable components of the reaction medium; and (3) a cofactor-independent ortho-hydroxylation reaction similar to polyphenol oxidase cresolase activity to which no variable components were added since the co-substrate O_2_ was present in the reaction medium as dissolved oxygen (Figure 1A–C and Table 1). An additional control enzymatic reaction containing metabisulfite as a variable component was also tested. Table 1 shows the specific activity in µmol·h^−1^·mg protein^−1^ obtained for the different tests and samples. The highest specific activity observed in the crude extract, particulate and soluble fraction was for PPO activity, while the other activities were either basal or did not show significant differences between them. The determination of the overall activity yield of the PPO in the soluble fraction was 61.54% with total PPO activity of 66.15 EU of the total of 107.42 EU present in the crude extract, while in the particulate fraction, the yield was 3.71% with a total PPO activity of 3.71 EU. Therefore, we carried out a partial purification of the soluble fraction by precipitation with ammonium sulphate, dialysis and ion exchange chromatography. For the soluble fraction precipitated with 80% ammonium sulphate and the chromatographic fractions of A5–A10, the three possible enzymatic activities of the hydroxylation of resveratrol mentioned above were also analyzed, and the specific activity is also shown in Table 1. The results show important PPO activity, especially in the chromatographic fractions A6–A9, with fraction A8 being where the highest value was found with 484-fold greater activity compared to basal activity in the presence of metabisulfite. Additionally, a certain level of 2-oxoglutarate-dependent dioxygenase (2-OGDD) activity could be observed in the chromatographic fractions A8 and A9 with 1.6-fold greater activity compared to basal activity in the absence of 2-oxoglutarate necessary for dioxygenase activity and the presence of metabisulfite. However, NADPH-dependent hydroxylation activity by cytochrome P450 was not detected.

The role of metabisulfite in the reaction medium was to inhibit the PPO activity when other enzymes were assayed. Likewise, the presence of ascorbate would favour the accumulation of an *o*-diphenol product, which, in the presence of PPO and oxygen, would undergo oxidation to the corresponding *o*-quinone. In fact, when ascorbate was omitted, the reaction medium turned orange, indicating the formation of *o*-quinone. As ascorbate is a natural antioxidant playing a central role in the redox homeostasis of plant cells [35], we assumed that our reaction medium would reliably reflect the cellular conditions favouring the accumulation of piceatannol, as observed on elicited grapevine cells [16].

Based on the results of the enzymatic assays performed, the reaction most likely to lead to a significant accumulation of piceatannol in elicited grapevine cells is that catalyzed by a PPO in the presence of ascorbic acid.

PPO is a bifunctional copper-containing metalloprotein that leads the regio-selective *o*-hydroxylation of monophenols to *o*-diphenols (cresolase activity) and the oxidation of *o*-diphenols to *o*-quinone (catecholase activity) (Figure 1C). The presence of a specific substrate, monophenolic or o-diphenolic compounds, promotes specific enzymatic activity. Treatment with the phytohormone MeJA has been shown to induce PPO activity and mRNA levels in tomato (*Solanum lycopersicum*) [36] and tea (*Camelia sinensis*) [37]. This phytohormone, together with MBCD, is not only responsible for triggering the large synthesis and extracellular accumulation of resveratrol [25,26], a monophenolic phytochemical that can be a substrate for the hydroxylation reaction, but it also may be responsible for increasing enzyme levels, leading to the detection of intracellular piceatannol in grapevine cell cultures at 120 h of elicitation with MBCD and MeJA [16].

### 3.2. Detection and Identification of PPO in Samples Catalysing the Synthesis of Piceatannol

**Figure 2:**
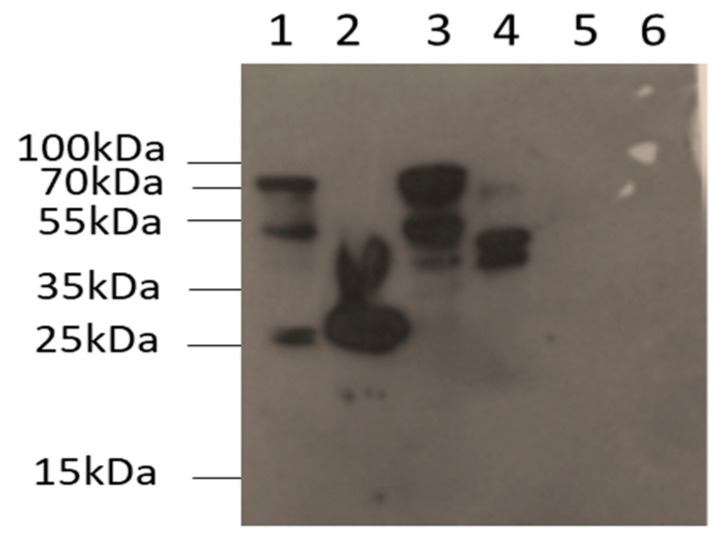
Western blot analysis in different fractions to confirm the presence of PPO protein with antisera PPO loquat [33]. 1. Crude extract, 2. particulate fraction, 3. soluble fraction after saturation with 80% ammonium sulphate, 4. A7–A9 chromatographic fractions, 5. A1–A5 chromatographic fractions, 6. A11, A12 chromatographic fractions.

The presence of PPO in both the crude extract and the different fractions was determined using Western blots (see Figure 2). The anti-loquat (*Eriobotrya japonica*) PPO antiserum cross-reacted with several bands in the grapevine cells’ crude extract, three intense at 25, 52 and 70 kDa and one faint at 40 kDa. When the crude extract was separated into particulate and soluble fractions, the antiserum reacted with two intense bands at 25 and 40 kDa in the particulate fraction and three bands, one at 40 of medium intensity and two intense at 52 and 70 kDa in the soluble fraction. In the chromatographic fractions, immunoreactive bands were only detected in the mixture of A7–A9 and were not found in A1–A5, A11 and A12. The antiserum reacted with three bands, two intense at 40 and 52 and one faint at 70 kDa. The presence of immunoreaction in the analyzed samples correlates with the PPO enzymatic activity observed and supports the presence of several PPO isoforms in grapevine cell cultures when stimulated.

Moreover, all chromatographic fractions were processed by tryptic in-liquid digestion and analyzed by liquid chromatography–tandem mass spectrometry with the aim of confirming the presence of PPO as a protein involved in the formation of piceatannol. Table S1 shows the PPO hit list for the A6–A9 chromatographic fractions, indicating the number of matching peptides found by MS/MS spectrum search and the percentage of sequence coverage with respect to the PPO-assigned protein and peptide sequences found in each chromatographic fraction. The PPO is clearly identified for A6–A9 chromatographic fractions, but not its respective isoform. To gain insight into the biological meaning of the results obtained, the complete sequences of four PPO proteins found in the databases for *V. vinifera* were aligned, and then, the peptides which were identified for each chromatographic fraction were mapped on them, as shown in Figure S1. As shown in Table 2, many peptide sequences were found to be exclusive of two protein sequences (QID41594.1, XP_010647098.3). However, in addition to the peptides common to other isoforms, there was one peptide (DFTDPDWLDAGFVFYDENAQLVR) unique to XP_010647098.3, providing conclusive evidence that this PPO isoform was in the PPO-active samples, and thus, it is the isoform most likely involved in the resveratrol hydroxylation activity reaction, although the presence and participation of other isoforms such as QID41594.1 cannot be ruled out.

The full-length grapevine PPO genes encode polypeptides between 602 and 624 amino acids accounting for Mw from 67.1 to 69.8 kDa. The sequence analysis with the TargetP 2.0 tool (https://services.healthtech.dtu.dk/services/TargetP-2.0/ (accessed on 7 August 2024)) used to detect targeting peptides in different subcellular compartments resulted in detection with a high probability of thylakoid luminal transfer peptides for QID41594.1, XP_059596123.1 and NP_001268045.1, but not for XP_010647098.3 (Supplementary file TP20_output_data). The sequence of the putative transfer peptide is highlighted in Figure S1. The hypothetical mature polypeptides would have between 554 and 555 amino acids with an Mw between 61.6 and 62 kDa.

Taken together, the results of Western blot detection, LC-MS-based PPO identification and sequence analysis show that the presence of a ca. 70 kDa band is consistent with the expected size of the non-processed polypeptide obtained from the soluble fraction and active chromatographic fractions thereof (Figure 2, lanes 3 and 4), whilst the presence of polypeptides of 40 kDa and smaller in the particulate fraction does not match the expected sized of ca. 62 kDa. Also, in addition to the expected polypeptide of 70 kDa, other polypeptides of smaller size (ca. 40 and 52 kDa) are detected in the soluble fraction and especially in the highly active chromatographic fractions. In addition to the cleavage process undergone by the full-size PPO polypeptide for translocation to thylakoid lumen that leads to a 5 kDa N-terminal peptide loss [38], other proteolytic processes have been described for plant PPO. In grapevine berry [39], loquat fruit [40] and lettuce (*Lactuca sativa*) leaf [41], among others, the native enzyme was extracted in a latent state of low activity. It has been shown that the enzyme can be reversibly activated by treatment with anionic detergents or pH changes [33] but also irreversibly activated by a trypsin proteolytic treatment in which the enzyme losses a ca. 20 kDa fragment [42]. Although no specific peptidases for in vivo PPO activation have been described to date, solid evidence for a self-cleavage mechanism of the isoform PPO1 from *Malus domestica* has been reported, which, however, does not occur in the isoenzyme PPO2 [43]. This recent discovery, besides the evidence of tryptic activation of grapevine PPO [42] and detection in highly active chromatographic fractions of bands with Mw losses of at least 20 kDa, suggest that there may be an in vivo proteolytic activation process for some isoforms of grapevine PPO.

Although the concept that several PPO isoforms are present in piceatannol production-catalyzing samples cannot be discarded with the present data, the isoform XP_010647098.3 accumulates more evidence for being responsible for that role.

### 3.3. Kinetic Characterization of Cresolase Activity of Vitis PPO on Resveratrol

**Figure 3:**
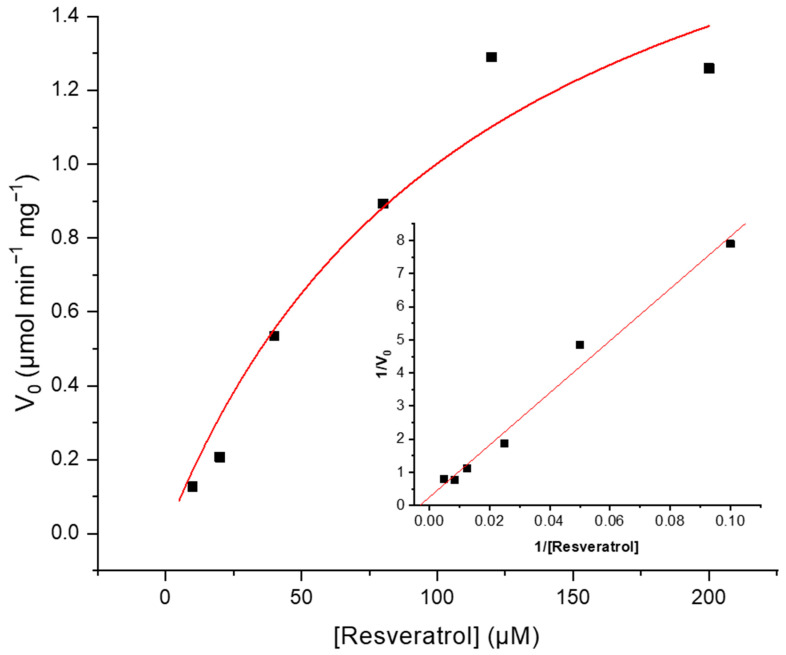
Steady-state kinetic analysis of enzymatic activity PPO from *Vitis vinifera*. Michaelis–Menten and Lineweaver–Burk (inset) curves of initial reaction rate versus resveratrol concentration. Equations and parameters obtained were V_0_ = Vmax × [S]/(Km + [S]), Vmax = 2.18 ± 0.46 µmol·min^−1^·mg^−1^, Km = 118.35 ± 49.84 µM and R = 0.96; y = 78.81x + 0.25 and R = 0.99.

The kinetic parameters of maximal velocity (Vmax) and Michaelis–Menten constant (Km) of resveratrol hydroxylation were studied using a mixture of A7 and A8 chromatographic fractions. Previously, a reaction mixture containing 200 µM of resveratrol was sampled periodically to assess the reaction progress and to set the incubation time of linear accumulation, which was established in 90 min, and thus used for the kinetic characterization. As shown in Figure 3, where Michaelis–Menten and Lineweaver–Burk (inset) plots are displayed, enzyme fractions exhibited Michaelian kinetics with respect to the substrate resveratrol. With respect to the kinetic parameters determined from the double reciprocal plots, the Km value is 118.35 ± 49.84 µM and the Vmax value is 2.18 ± 0.46 µmol·min^−1^·mg^−1^. The Km value was compared with other Km values reported in the literature for the PPO activity of grapes both for diphenolic and monophenolic substrates. Most substrates tested with grape PPO reported in the literature are diphenols exhibiting Km values in the millimolar range, while the monophenol p-cresol showed a Km value below millimolar (see Supplementary Information, Table S2). This compares very well with the submillimolar Km for resveratrol, which undergoes hydroxylation on its monophenolic ring.

## 4. Conclusions

Based on the work presented here, it can be concluded that the enzyme responsible for the o-hydroxylation of resveratrol for the biosynthesis of piceatannol in grapevine cell cultures elicited with MBCD and MeJA is the cresolase activity of PPO, whilst other hydroxylating reactions tested are quite unlikely to be responsible. Based on the number and uniqueness of peptides detected in trypsin-digested PPO-active samples, the most likely isoform of PPO responsible for this function is XP_010647098.3. The 69.8 kDa protein encoded a lacks of known chloroplast targeting peptides, consistent with the detection of the ca. 70 kDa immunoreactive band, but the detection of 52 and 40 kDa bands in highly active fractions suggests that proteolytic processing is a key mechanism for the catalytic action of the enzyme. Therefore, among the possible pathways hypothesized for the biosynthesis of piceatannol from resveratrol in grapevine, the most likely is that catalyzed by PPO cresolase activity depicted in Figure 1C. This report represents the first elucidation of the piceatannol biosynthetic pathway. Further studies will be necessary to fully characterize the biosynthetic pathway in planta.

## Figures and Tables

**Table 1:** Specific activity was obtained in different reactions with samples and fractions obtained from grapevine cell cultures elicited with 50 mM MBCD and 100 µM of MeJA. Data are the mean of two independent replicates ± SD.

REAGENT	CYP (-PPO)	OGDD (-PPO)	Basal (-PPO)	PPO
NADPH	✓	Ø	Ø	Ø
OxoGlutarate	Ø	✓	Ø	Ø
Metabisulfite	✓	✓	✓	Ø
Ascorbic Acid	✓	✓	✓	✓
Sample/Fraction	µmol/h/mg	µmol/h/mg	µmol/h/mg	µmol/h/mg
Control (-)	n.d.	n.d.	n.d.	n.d.
Crude Extract	0.019 ± 0.002	0.017 ± 0.004	0.025 ± 0.005	0.231 ± 0.037
Soluble Fract	0.018 ± 0.007	0.025 ± 0.003	0.019 ± 0.001	0.147 ± 0.058
Particulate Fract	0.042 ± 0.09	0.018 ± 0.006	0.059 ± 0.021	0.247 ± 0.089
80% (NH_4_)_2_ SO_4_	0.068 ± 0.06	0.099 ± 0.025	0.073 ± 0.038	0.343 ± 0.017
A5	0.030	n.d	0.054	1.813
A6	0.113	0.135	0.171	101.48
A7	0.315	0.936	0.803	661.04
A8	0.571	3.776	2.130	1031.25
A9	0.760	3.550	2.296	453.79
A10	0.273	0.275	0.299	14.83

n.d.: not detected; the symbols (✓) and (Ø) stand for reagent added or not added to the assay medium, respectively.

**Table 2:** Summary of tryptic peptides identified among the different PPO-active samples analyzed by LC-MS/MS and database search confidently assigned to full-length grapevine PPO isoforms.

Tryptic Peptide Identified	PPO Isoform			
	XP_010647098.3	QID41594.1	XP_059596123.1	NP_001268045.1
AIELMK	✓	✓		
ALPDDDPR	✓	✓	✓	✓
LIDLDYNLTDSNDTNEQQISSNLSIMYR	✓	✓		
TTSLFMGAAYR	✓	✓		
DPIFFSHHSNVDR	✓	✓		
DFTDPDWLDAGFVFYDENAQLVR	✓			
IGISELLEDLEAEDDDSVVVTLVPR	✓	✓		

## Data Availability

Data are contained within the article and Supplementary Materials.

## Supplementary Materials

The following supporting information can be downloaded at https://www.mdpi.com/article/10.3390/plants13182602/s1: Additional supporting information may be found in the online version of this article: Table S1. Total identifications of PPO proteins. Table S2. Km values for the activity of vine PPOs with different substrates, diphenols and monophenols. Figure S1. Mapping of identified peptides on multiple sequence alignment of grapevine PPOs. The sequences of the peptides identified by LC-MS/MS are marked in red. The thylakoid signal peptide sequence is shaded in black. Supplementary file TP20_output_data. Thylakoid peptide analysis. References [44,45,46,47,48,49] are cited in the supplementary materials.

## Abbreviation

2-OGDD	2-oxoglutarate-dependent dioxygenase
CYP	cytochrome P450 hydroxylase/monoxygenase
EU	enzyme units
HEPES	2-[4-(2-hydroxyethyl)piperazin-1-yl]ethanesulfonic acid
MBCD	dimethyl-β-cyclodextrin
MeJA	methyl jasmonate
MRM	multiple reaction monitoring
PMSF	phenylmethylsulfonyl fluoride
PPO	polyphenol oxidase
PVPP	polyvinylpolypyrrolidone
STS	stilbene synthase
TCA	trichloroacetic acid
*t*-R	*trans*-resveratrol (3,4′,5-trihydroxy-*trans*-stilbene)
