# Supplementary material for: Biosynthesis of Piceatannol from Resveratrol in Grapevine Can Be Mediated by Cresolase-Dependent Ortho-Hydroxylation Activity of Polyphenol Oxidase
Source: Plants (Basel). 2024 Sep 18;13(18):2602. doi: 10.3390/plants13182602 (PMC11434850; doi:10.3390/plants13182602)
Supplement: Supplementary file 1 [file plants-13-02602-s001.zip › Figure S1.pdf]

```
QID41594.1          -----------------------MASLSPPATTTTTTNISSLQPSPLFHKASQVSLVGKR   37
XP_010647098.3      MSVHYKRKEGPRPFSYPYRLISTMASLSPPAT-TTTTNISSLQPSPLFHKASQVSLVGKR   59
XP_059596123.1      -------------------MASLPWSLTTSTAIANTTNISALPPSPLFHRVSHVPIARNR   41
NP_001268045.1      -------------------MASLPWSLTTFTAIANTTNISAFPPSPLFQRASHVPVARNR   41
                                             **:   :: :.*****:: *****::.*:* :. :*

QID41594.1          NPRFVRRVVSCNATNGDERSTSSSKSGESPLGKLDRRNVLLGLGGLYGV-SGLGAADPFA   96
XP_010647098.3      NPRFVRRVVSCNATNGDERSTSSSKSGESPLGKLDRRNVLLGLGGLYGV-SGLGAADPFA   118
XP_059596123.1      SRRFAPSKVSCNAANGDPNSDSTSDIRETSPGKLDRRNVLLGIGGLYGAAAGLGATKPLA   101
NP_001268045.1      SRRFAPSKVSCNSANGDPNSDSTSDVRETSSGKLDRRNVLLGIGGLYGAAGGLGATKPLA   101
                    . **.   ****::*** .* *:*.  *:  ***********:*****. .****:.*:*

QID41594.1          FAAPISPPDLSECGAADLPAGAQPTNCCPPVSSKIIDFKLLPKNSPLRIRPAAHLASKEY   156
XP_010647098.3      FAAPISPPDLSECGAADLPAGAQPTNCCPPVSSKIIDFKLPPKNSPFRIRPAAHLASKEY   178
XP_059596123.1      FGAPIQAPDISKCGTATVPDGVTPXNCCPPVTTKIIDFQLPSSGSPMRTRPAAHLVSKEY   161
NP_001268045.1      FGAPIQAPDISKCGTATVPDGVTPTNCCPPVTTKIIDFQLPSSGSPMRTRPAAHLVSKEY   161
                    *.***. **:*:**:* :* *. * ******::*****:*  ..**:* ******.****

QID41594.1          IAKYKKAIELMKALPDDDPRSFMQQADVHCAYCNGAYHQVGFPDLDLQVHNSWLFLPYHR   216
XP_010647098.3      IAKYKKAIELMKALPDDDPRSFMQQADVHCAYCNGAYHQVGFPDLDLQVHNSWLFLPYHR   238
XP_059596123.1      LAKYKKAIELQKALPDDDPRSFKQQANVHCTYCQGAYDQVGYTDLELQVHASWLFLPFHR   221
NP_001268045.1      LAKYKKAIELQKALPDDDPRSFKQQANVHCTYCQGAYDQVGYTDLELQVHASWLFLPFHR   221
                    :********* *********** ***:***:**:***.***: **:**** ******:**

QID41594.1          YYLYFYEKILGKLINDPTFALPFWNWDAPAGMQMPAMFADPNSPLYDKLRDAKHQPPKLI   276
XP_010647098.3      YYLYFYEKILGKLINDPTFALPFWNWDAPAGMQMPAMFADPNSPLYDKLRDAKHQPPKLI   298
XP_059596123.1      YYLYFNERILAKLIDDPTFALPYWAWDNPDGMYMPTIYASSPSSLYDEKRNAKHLPPTVI   281
NP_001268045.1      YYLYFNERILAKLIDDPTIALAYWAWDNPDGMYMPTIYASSPSSLYDEKRNAKHLPPTVI   281
                    ***** *:**.***:***:** :* ** * ** **:::*.  * ***: *:*** **.:*

QID41594.1          DLDYNLTDSNDTNEQQISSNLSIMYRQIVSSAKTTSLFMGAAYRAGDEPDPGQGTLENIP   336
XP_010647098.3      DLDYNLTDSNDTNEQQISSNLSIMYRQIVSSAKTTSLFMGAAYRAGDEPDPGQGTLENIP   358
XP_059596123.1      DLDYDGTESTIPDDELKTDNLAIMYKQIVSGATTPKLFLGYPYRAGDAIDPGAGTLEHVP   341
NP_001268045.1      DLDYDGTEPTIPDDELKTDNLAIMYKQIVSGATTPKLFLGYPYRAGDAIDPGAGTLEHVP   341
                    ****: *: .  :::  :.**:***:****.*.* .**:*  *****  *** ****::*

QID41594.1          HGPVHIWCGDRTQPNIEDMGNFYSAARDPIFFSHHSNVDRMWTVWKGLGGK-RKDFTDPD   395
XP_010647098.3      HGPVHIWCGDRTQPNIEDMGNFYSAARDPIFFSHHSNVDRMWTVWKGLGGK-RKDFTDPD   417
XP_059596123.1      HNIVHKWTGLADKP-SEDMGNFYTAGRDPIFFGHHANVDRMWNIWKTIGGKNRKDFTDTD   400
NP_001268045.1      HNIVHKWTGLADKP-SEDMGNFYTAGRDPIFFGHHANVDRMWNIWKTIGGKNRKDFTDTD   400
                    *. ** * *   :*  *******:*.******.**:******.:** :*** ****** *

QID41594.1          WLDAGFVFYDENAQLIRVKVRDCLDPTKLRYAYQEVDIPWLKSRPKPRQILKKVVNKLKH   455
XP_010647098.3      WLDAGFVFYDENAQLVRVKVRDCLDPTKLRYAYQEVDIPWLKSRPKPRQILKKVVNKLKH   477
XP_059596123.1      WLDATFVFYDENKQLVKVKVSDCVDTSKLRYQYQDIPIPWLPKNTKAKAK----TTTKSS   456
NP_001268045.1      WLDATFVFYDENKQLVKVKVSDCVDTSKLRYQYQDIPIPWLPKKTKAKAK----TTTKSS   456
                    **** ******* **::*** **:* :**** **:: ****  .. * :      ... .

QID41594.1          KSGVANAAEIPSS---SSIVFPIKLDSTLRVDVPRPRKSRSKKEKEDEEEVLVIKGIELE   512
XP_010647098.3      KSGVANAAEIPSS---SSIVFPIKLDSTLRVDVPRPRKSRSKKEKEDEEEVLVIKGIELE   534
XP_059596123.1      KSGVAKAAELPKTTISSIGDFPKALNSVIRVEVPRPKKSRSKKEKEDEEEVLLIKGIELD   516
NP_001268045.1      KSGVAKAAELPKTTISSIGDFPKALNSVIRVEVPRPKKSRSKKEKEDEEEVLLIKGIELD   516
                    *****:***:*.:    *    **  *:*.:**:****:***************:******:

QID41594.1          RDKFVKFDIFINDEDDPVSRPDNTEFAGSFVNVPHQHSHGKKKNTILRIGISELLEDLEA   572
XP_010647098.3      RDKFVKFDIFINDEDDPVSRPDNTEFAGSFVNVPHQHSHGKKKNTILRIGISELLEDLEA   594
XP_059596123.1      RENFVKFDVYINDEDYSVSRPKNSEFAGSFVNVPHKHMKEMKTKTNLRLAINELLEDLGA   576
NP_001268045.1      RENFVKFDVYINDEDYSVSRPKNSEFAGSFVNVPHKHMKEMKTKTNLRFAINELLEDLGA   576
                    *::*****::*****  ****.*:***********:*  :  *.:* **:.*.****** *

QID41594.1          EDDDSVVVTLVPRYGADVITIGGIEIELAS- 602
XP_010647098.3      EDDDSVVVTLVPRYGADVITIGGIEIELAS- 624
XP_059596123.1      EDDESVIVTIVPRAGGDDVTIGGIEIEFVSD 607
NP_001268045.1      EDDESVIVTIVPRAGGDDVTIGGIEIEFVSD 607
                    ***:**:**:*** *.* :********:.*
```

Figure S1. Mapping of identified peptides on multiple sequence alignment of grapevine PPOs. The sequence of the peptides identified by LC-MS/MS are marked in red. The thylakoid signal peptide sequence is shaded in black.
