# Supplementary material for: Biosynthesis of Piceatannol from Resveratrol in Grapevine Can Be Mediated by Cresolase-Dependent Ortho-Hydroxylation Activity of Polyphenol Oxidase
Source: Plants (Basel). 2024 Sep 18;13(18):2602. doi: 10.3390/plants13182602 (PMC11434850; doi:10.3390/plants13182602)
Supplement: Supplementary file 1 [file plants-13-02602-s001.zip › plants-3176159-supplementaryTable S2_revAUTH.pdf]

Table S2. Km values for activity of vine PPOs with different substrates, diphenols and monophenols.

**Substrate**	K_M **(mM)**	***Reference***
Resveratrol	0.118	This work.
4-methylcatechol	22	[44]
guaiacol	32.2	[44]
pyrogallol	32.2	[44]
catechin	5.2	[44]
epicatechin	5.2	[44]
4-methylcatechol	5.0 ± 0.3 mM	[45]
Dopamine	16.1	[46]
Tyramine	7.7	[46]
4-methylcatechol	9	[39]
4-methylcatechol *Vitis labruscana* Bailey × *Vitis vinifera* L.	1.4	[47]
4-methylcatechol	4.8	[48]
Catechol	18.33	[48]
Catechol	156.9	[49]
4-Methylcatechol	103.4	[49]
Pyrogallol	227.1	[49]
Caffeic acid	0.82	[49]
Catechol	143.69	[49]
4-Methylcatechol	89.00	[49]
Pyrogallol	72.14	[49]
Caffeic acid	0.64	[49]
p-cresol	0.5	[39]
